# Cytochrome P450 CYP3A in human renal cell cancer

**DOI:** 10.1038/sj.bjc.6690292

**Published:** 1999-04

**Authors:** G I Murray, M C E McFadyen, R T Mitchell, Y-L Cheung, A C Kerr, W T Melvin

**Affiliations:** Departments of Pathology and Molecular and Cell Biology, University of Aberdeen, Aberdeenl, Foresterhil, AB25 2ZD, UK

**Keywords:** cytochrome P450, kidney, neoplasm

## Abstract

Renal cell cancer is the main malignant tumour of the kidney and has an increasing incidence. This type of tumour has a poor prognosis and shows intrinsic resistance to several anti-cancer drugs. The CYP3A P450 family, which consists of three closely related forms, is involved in the oxidative activation and deactivation of a variety of carcinogens and several anti-cancer drugs. In this study the presence and cellular localization of CYP3A has been investigated using a combination of immunohistochemistry, immunoblotting and reverse transcriptase polymerase chain reaction (RT-PCR) in renal cell cancer and corresponding normal kidney. CYP3A was consistently expressed in both renal call cancer and in normal kidney. In renal cell cancer, CYP3A was localized to tumour cells and in normal kidney the predominant cellular localization of CYP3A was to proximal tubular epithelial cells. RT-PCR showed that both CYP3A5 mRNA and CYP3A7 mRNA were consistently present in both tumour and normal samples, while CYP3A4 mRNA was present in 65% of tumours and 90% of normal samples. This study indicates that individual members of the CYP3A family are expressed in renal cell cancer. The presence of CYP3A in renal cell cancer might be important in the metabolic potentiation as well as the detoxification of chemotherapeutic agents used to renal cancer. © 1999 Cancer Research Campaign


*British Journal of Cancer* (1999) **79**(11/12), 1836–1842
© 1999 Cancer Research Campaign
Article no. bjoc.1998.0292

### Cytochrome P450 CYP3A in human renal cell cancer

**GI Murray, MCE McFadyen, RT Mitchell, Y-L Cheung, AC Kerr and WT Melvin**

Departments of Pathology and Molecular and Cell Biology, University of Aberdeen, Foresterhill Aberdeen, AB25 2ZD, UK

**Summary** Renal cell cancer is the main malignant tumour of the kidney and has an increasing incidence. This type of tumour has a poor prognosis and shows intrinsic resistance to several anti-cancer drugs. The CYP3A P450 family, which consists of three closely related forms, is involved in the oxidative activation and deactivation of a variety of carcinogens and several anti-cancer drugs. In this study the presence and cellular localization of CYP3A has been investigated using a combination of immunohistochemistry, immunoblotting and reverse transcriptase polymerase chain reaction (RT-PCR) in renal cell cancer and corresponding normal kidney. CYP3A was consistently expressed in both renal call cancer and in normal kidney. In renal cell cancer, CYP3A was localized to tumour cells and in normal kidney the predominant cellular localization of CYP3A was to proximal tubular epithelial cells. RT-PCR showed that both CYP3A5 mRNA and CYP3A7 mRNA were consistently present in both tumour and normal samples, while CYP3A4 mRNA was present in 65% of tumours and 90% of normal samples. This study indicates that individual members of the CYP3A family are expressed in renal cell cancer. The presence of CYP3A in renal cell cancer might be important in the metabolic potentiation as well as the detoxification of chemotherapeutic agents used to renal cancer.

**Keywords:** cytochrome P450; kidney; neoplasm

Renal cell cancer is the commonest malignant tumour of the adult kidney accounting for about 85% of malignant kidney tumours and the incidence of this tumour is steadily rising (Stadler and Vogelzang, 1993; Motzer et al, 1996). The aetiology and pathogenesis of renal cell cancer is not fully understood, although several environmental risk factors have been proposed, including smoking, occupational exposure to heavy metals (e.g. cadmium and arsenic) and obesity. The majority of renal cell cancers are considered to develop from proximal tubular epithelium. Renal cell cancer also responds poorly to wide variety of anti-cancer drugs including vinca alkaloids, etoposide, anthracyclines, taxanes and mitomycin-C (Chapman and Goldstein, 1995; Motzer et al, 1996). Furthermore, agents such as cyclosporin which reverse P-glycoprotein-associated multidrug resistance have not been shown to enhance anti-tumour activity in renal cell cancer (Yagoda et al, 1995).

The cytochromes P450 (P450s) are a very large gene family of constitutive and inducible haem containing enzymes. The P450s are classified into families, sub-families and individual forms according to gene sequence homology (individual P450s are identified by the prefix CYP and current P450 nomenclature is outlined in Nelson et al, 1996). There are two broad groups of mammalian P450s. A large group of P450s whose primary role is the oxidative activation and/or deactivation of a wide variety of xenobiotics (CYP1, CYP2, CYP3) while there is a much smaller group of P450s which are constitutively expressed in endocrine glands (adrenal gland, ovary testis) where they are specifically involved in steroid hormone synthesis. Those P450s that have been primarily characterized according to their ability to metabolize xenobiotics are also capable of metabolizing endogenous compounds especially eicosanoids (Capdevila et al, 1992) and steroid hormones (Sarabia et al, 1997) and thus those P450s may also have endogenous functions particularly involvement in cell regulation and cell signalling (Nebert, 1994).

The P450s are considered to have a central role in chemical carcinogenesis and are involved in tumour initiation and promotion as they can activate or deactivate many carcinogens (Kawajiri and Fujii-Kuriyama, 1991; Guengerich 1992; Gonzalez and Gelboin, 1994). Furthermore, the P450s can influence the response of established tumours to anti-cancer drugs as P450s are involved in the metabolism of several anti-cancer drugs (Kivistö et al, 1995*a*).

The CYP3A P450 family is one of the main P450 families involved in xenobiotic metabolism including the metabolism of various carcinogens (Gonzalez and Gelboin, 1994; Roberts-Thomson et al, 1995) and several current anti-cancer drugs (Kivistö et al, 1995*a*). This family of P450s consists of three closely related forms: CYP3A4, CYP3A5 and CYP3A7. CYP3A4 is the major form of P450 which is constitutively expressed in liver whereas CYP3A5 is only found in a minority of liver samples. However, CYP3A5 appears to show a more widespread constitutive expression in extrahepatic tissues while CYP3A7 is the main form of P450 found in fetal liver (Kitada and Kamataki, 1994).

Several studies have identified the presence of CYP3A in tumours, including breast cancer (Murray et al, 1993), colon cancer (Massaad et al, 1992), lung cancer (Kivistö et al, 1995*b*) and stomach cancer (Murray et al, 1998) however, no studies to date have investigated the presence of CYP3A in renal cancer. In this study the presence and localization of CYP3A has been investigated by a combination of immunohistochemistry, immunoblotting and reverse transcriptase polymerase chain reaction (RT-PCR) in primary renal cell cancer and normal human kidney.

*Received 19 May 1998*
*Revised 20 August 1998*
*Accepted 26 August 1998*

*Correspondence to:* GI Murray

### MATERIALS AND METHODS

### Tissue

Paired samples of renal cancer and corresponding normal kidney were obtained from 20 adult patients (15 male, five female) undergoing surgery for primary renal cancer and submitted to the Department of Pathology, University of Aberdeen, for diagnosis. None of the patients had received chemotherapy prior to surgery. All the tumours were primary renal cell cancers and histological classification of the tumours performed according to current criteria (Kovacs et al, 1997) showed 17 clear cell carcinomas, two chromophobe carcinomas and one papillary carcinoma. The clinicopathological data for each patient are summarized in Table 1.

When the tissue samples were prepared they were carefully selected and dissected to ensure no contamination by connective tissue or fat and the tumour samples were selected from areas of macroscopically viable tumour. Areas of necrotic tumour or haemorrhage were avoided. As far as possible, the tumour samples were selected from areas of viable tumour of high tumour cellularity (confirmed by microscopy of haematoxylin and eosin stained section) and contained only a very small proportion of non-tumour cells (either inflammatory cells or stromal cells). This is important when interpreting the results of any investigation that involves homogenization of tumours, since tumour samples can consist of a variable proportion of tumour and non-tumour cells. Furthermore, any adjacent normal renal tissue was also removed from tumour samples prior to sample preparation.

Small blocks of normal and tumour kidney were fixed in 10% neutral-buffered formalin and embedded in wax for histological diagnosis and immunohistochemistry. Samples of both normal and viable tumour kidney were also frozen in liquid nitrogen for protein and RNA analysis and stored at –75°C prior to use.

**Table 1** Clinicopathological details of patients

| Case no. | Age | Sex | Histopathology of renal cancer | TNM stage |
|---|---|---|---|---|
| 1 | 67 | M | Clear cell | 2 |
| 2 | 63 | M | Chromophobe | 2 |
| 3 | 60 | M | Clear cell | 3 |
| 4 | 48 | F | Chromophobe | 2 |
| 5 | 78 | F | Clear cell | 2 |
| 6 | 59 | M | Clear cell | 3 |
| 7 | 52 | M | Clear cell | 3 |
| 8 | 39 | F | Clear cell | 3 |
| 9 | 72 | F | Clear cell | 3 |
| 10 | 88 | M | Clear cell | 3 |
| 11 | 68 | M | Papillary | 2 |
| 12 | 66 | M | Clear cell | 3 |
| 13 | 42 | M | Clear cell | 2 |
| 14 | 71 | M | Clear cell | 2 |
| 15 | 78 | M | Clear cell | 2 |
| 16 | 82 | M | Clear cell | 2 |
| 17 | 58 | M | Clear cell | 2 |
| 18 | 39 | F | Clear cell | 2 |
| 19 | 45 | M | Clear cell | 3 |
| 20 | 63 | M | Clear cell | 2 |

M, male; F, female.

### Immunohistochemistry

CYP3A immunoreactivity was identified using a monoclonal antibody (HL3) which recognizes CYP3A4, CYP3A5 and CYP3A7 (Murray et al, 1987, 1988). Sections (4 μm in thickness) of normal and tumour kidney were dewaxed in xylene, rehydrated in alcohol and washed in 0.05 M Tris-HCl pH 7.6 containing 150 mM sodium chloride (TBS). The primary antibody was applied as undiluted tissue culture supernatant for 60 min at room temperature. Rabbit anti-mouse immunoglobulin (1/100, Dako, High Wycombe, UK) and monoclonal APAAP (1/100, Dako) were subsequently applied (McKay et al, 1995). Following incubation with each antibody the sections were washed for three 5-min periods in TBS to remove unbound antibody. Sites of bound alkaline phosphatase were demonstrated colorimetrically using a solution containing 3 mg bromo-chloro-indolyl phosphate (Sigma, Poole, Dorset, UK), 10 mg nitro blue tetrazolium (Sigma), 6 mg sodium azide and 4 mg levamisole (Sigma) in 10 ml 0.05 M Tris-HCl buffer pH 9.0 containing 0.2% magnesium chloride ($MgCl_2$). After incubating the sections for 30 min at room temperature, the enzyme reaction was stopped by washing the sections for 5 min in cold tap water. The slides were then counterstained with haematoxylin, air-dried and mounted in glycerine jelly. The sections were examined using bright field light microscopy in order to establish the presence or absence of immunostaining, and its distribution. TBS in place of the primary antibody was used as a negative control while normal adult human liver which had been obtained from partial hepatectomy specimens and fixed in formalin was used as a positive control.

### Preparation of microsomes

Frozen samples of normal kidney and kidney tumour were thawed on ice in 0.01 M Tris-HCl pH 7.4 containing 1.15% potassium chloride. Once thawed the tissue was homogenized in 0.01 M Tris-HCl containing 0.25 M sucrose and 15% glycerol using a Polytron PT3000 homogenizer (Kinematica AG, Switzerland). The crude homogenates were centrifuged at 15 000 ***g*** for 20 min at 4°C using a Centrikon T-124 centrifuge (Kontron Instruments, Cumbernauld, UK). The resultant supernatants were then centrifuged at 180 000 ***g*** (44 000 rpm) for 1 h at 4°C using a Centrikon T-1160 centrifuge (Kontron Instruments). The pellet obtained after centrifugation was resuspended in 0.1 M Tris-HCl containing 15% glycerol and 1 mM EDTA (Sigma) and centrifuged again at 180 000 ***g*** (44 000 rpm) for 1 h at 4°C. The final microsomal pellet was resuspended in 0.1 M Tris-HCl containing 15% glycerol and 1 mM EDTA, and the microsomes were then stored at –75°C prior to use. The protein concentration of each sample of microsomes was determined using Bradford's method (Bradford, 1976).

### Immunoblotting

Microsomal proteins (30 μg of protein were loaded per lane for each kidney sample) were electrophoretically separated at constant current in a 10% polyacrylamide gel using a Hoefer SE600 electrophoresis system (Hoefer Pharmacia Biotechnology Inc., MN, USA) and then transferred at constant current for 18 h to nitrocellulose (Hybond ECL, Amersham Life Sciences, Little Chalfont, UK) by electroblotting using a Hoefer TE42 blotting system (Hoefer Pharmacia Biotechnology). Non-specific binding sites

© Cancer Research Campaign 1999

Table 2 Oligonucleotide primer sequences for CYP3A4, CYP3A5, CYP3A7 and β-actin

| P450 | Primer sequence (5′ to 3′) | Nucleotide location | Product size (bp) | GenBank accession no. |
|---|---|---|---|---|
| CYP3A4 | Forward CTGTGTGTTTCCAAGAGAAGTTTAC | 782– 805 | 742 | M14096 |
| | Reverse TGGTTGAAGAAGTCCTCCTAAG | 1523–1502 | | |
| CYP3A5 | Forward GTCTCTCTGTTTCCAAAAGATACC | 799– 822 | 737 | J04813 |
| | Reverse TGAAGAAGTCCTTGCGTGTC | 1535–1516 | | |
| CYP3A7 | Forward CTATGATACTGTGCTACAGT | 1041–1060 | 474 | D00408 |
| | Reverse TCAGGCTCCACTTACGGTCT | 1496–1515 | | |
| β-actin | Forward TGACGGGGTCACCCACACTGTGCCCATCTA | 1038–1067 | 661 | X00351 |
| | Reverse CTAGAAGCATTGCGGTGGACGATGGAGGG | 1876–1905 | | |

were blocked by incubation of the nitrocellulose membrane for 60 min at room temperature in wash buffer consisting of 2% non-fat milk (Marvel, Premier Beverages, Stafford, UK) in 10 mM phosphate-buffered saline (PBS) containing 0.05% Tween 20 (Sigma). The nitrocellulose was then sequentially incubated with anti-CYP3A antibody (1/1000) and goat anti-rabbit immunoglobulin conjugated to horseradish peroxidase (1/2000; Bio-Rad, Hemel Hempstead, UK). This polyclonal antibody also recognizes CYP3A4, CYP3A5 and CYP3A7 (Shaw et al, 1989) and is more effective by immunoblotting than the monoclonal antibody used for immunohistochemistry. After each antibody application the membrane was washed for five 10-min periods with the wash buffer and after removal of unbound secondary antibody the membrane was further washed in 10 mM PBS for five 10-min periods. Horseradish peroxidase was then demonstrated using an enhanced chemiluminescent technique (Amersham Life Sciences) which was performed as previously described (McKay et al, 1995; Murray et al, 1997).

Microsomes prepared from human lymphoblastoid cells which contained either expressed human CYP3A4 or CYP3A5 were used as positive controls and were obtained from Gentest Corp, Woburn, MA, USA.

### Isolation of RNA

Total RNA from kidney samples was isolated using RNAzol B (Biogenesis Ltd, Poole, Dorset, UK) used according to the manufacturer's instructions. The isolated RNA was resuspended in diethyl pyrocarbonate (DEPC, Sigma) treated water and quantified spectrophotometrically at 260 nm and 280 nm respectively. All the RNA samples had 260:280 ratios between 1.8 and 2.0.

### cDNA synthesis

Synthesis of cDNA was performed with a reverse transcriptase kit (Promega, Southampton, UK) using the following reaction conditions as recommended by Promega: 1 μg of RNA, 1 × reverse transcriptase buffer (10 mM Tris-HCl, pH 8.8, 50 mM potassium chloride and 0.1% Triton X-100), 5 mM $MgCl_2$, 1 mM of each dNTP, 0.5 unit ribonuclease inhibitor, 15 units of avian myeloblastosis reverse transcriptase and 0.5 μg oligo $dT_{15}$ primer in a final volume of 20 μl. Synthesis of cDNA was performed at 42°C for 60 min and the reaction was stopped by heating to 99°C for 5 min followed by chilling on ice. The cDNA was then stored at –75°C until required.

### PCR

PCR was carried out in 50-μl reaction volumes with the following reaction conditions: 2 μl of cDNA, 10 mM Tris-HCl (pH 8.3), 50 mM potassium chloride, 0.25 units of Amplitaq Gold (PE Applied Biosystems, Warrington, UK), 400 μM dNTP (Promega) and 2.5 mM $MgCl_2$ (β-actin), 3 mM $MgCl_2$ (CYP3A7) or 3.5 mM $MgCl_2$ (CYP3A4 and CYP3A5). The amount of each primer in the PCR reaction was 80 pmol for β-actin and 85 pmol for each P450 primer. All the P450 oligonucleotide primers were obtained from Life Technologies (Paisley, Renfrewshire, UK) while the β-actin oligonucleotide primers were purchased from Stratagene, Cambridge, UK. The oligonucleotide primer sequences, nucleotide location and expected product size for CYP3A4, CYP3A5, CYP3A7 and β-actin are detailed in Table 2. PCR with Amplitaq gold requires an initial precycling step at 95°C for 15 min to activate the enzyme prior to commencing the thermocycling. The following thermocycling conditions (as recommended by Stratagene) were used for β-actin: 94°C for 5 min, 60°C for 5 min followed by 40 cycles of amplification consisting of 72°C for 90 s, 94°C for 48 s, 60°C for 48 s and a final 72°C extension for 10 min. The thermocycling parameters for CYP3A4, CYP3A5 and CYP3A7 were: 40 cycles of amplification consisting of 94°C for 30 s, 60°C for 30 s and 72°C for 60 s, followed by a final 2-min extension at 72°C. Both negative and positive controls were included in each PCR reaction. The negative control was DEPC-treated water in place of the template cDNA. The positive controls for both CYP3A4 and CYP3A5 was cDNA prepared from total RNA isolated from normal adult human liver previously shown to contain CYP3A4 and CYP3A5 (McFadyen et al, 1998) while the positive control for CYP3A7 was cDNA prepared from total RNA isolated from human fetal liver (the gestational age of the fetus was 16 weeks). The PCR products (10 μl) were separated by gel electrophoresis using a 1.5% agarose gel, stained with ethidium bromide (Sigma) and visualized by trans-illumination with ultra-violet light. The gels were photographed with Polaroid type 665 black and white film.

### Sequencing of PCR products

Fluorescence-based DNA sequencing was performed to confirm the identity of the individual PCR products. PCR samples were purified for sequencing by using centricon C-100 columns (Amicon, Gloucester, UK) and approximately 150 ng of template were sequenced using a *Taq* dye deoxy terminator sequencing kit

© Cancer Research Campaign 1999

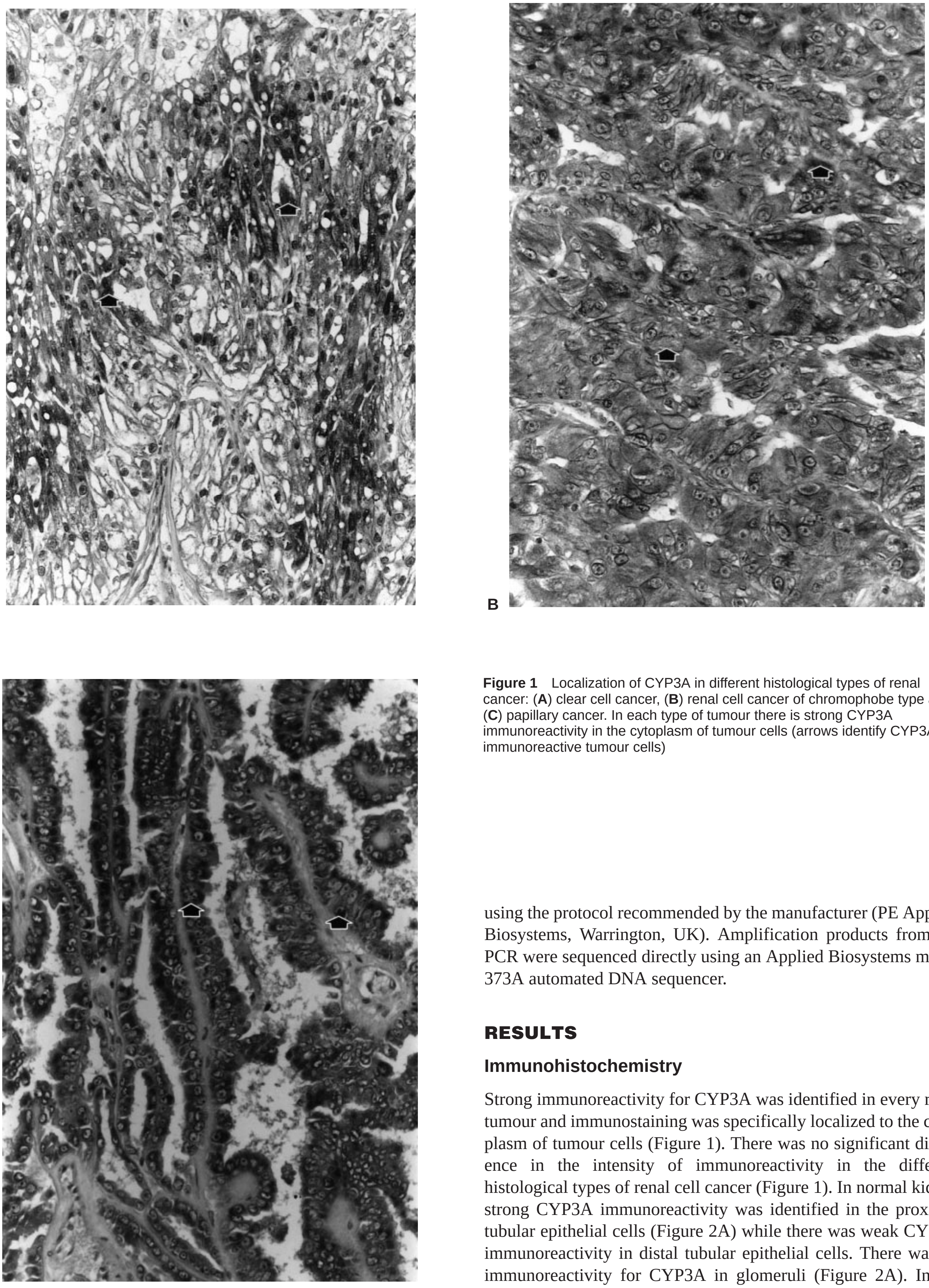

A B C

**Figure 1** Localization of CYP3A in different histological types of renal cancer: (**A**) clear cell cancer, (**B**) renal cell cancer of chromophobe type and (**C**) papillary cancer. In each type of tumour there is strong CYP3A immunoreactivity in the cytoplasm of tumour cells (arrows identify CYP3A immunoreactive tumour cells)

using the protocol recommended by the manufacturer (PE Applied Biosystems, Warrington, UK). Amplification products from the PCR were sequenced directly using an Applied Biosystems model 373A automated DNA sequencer.

### RESULTS

### Immunohistochemistry

Strong immunoreactivity for CYP3A was identified in every renal tumour and immunostaining was specifically localized to the cytoplasm of tumour cells (Figure 1). There was no significant difference in the intensity of immunoreactivity in the different histological types of renal cell cancer (Figure 1). In normal kidney strong CYP3A immunoreactivity was identified in the proximal tubular epithelial cells (Figure 2A) while there was weak CYP3A immunoreactivity in distal tubular epithelial cells. There was no immunoreactivity for CYP3A in glomeruli (Figure 2A). In the medulla there was strong staining in collecting duct epithelium (Figure 2B).

© Cancer Research Campaign 1999

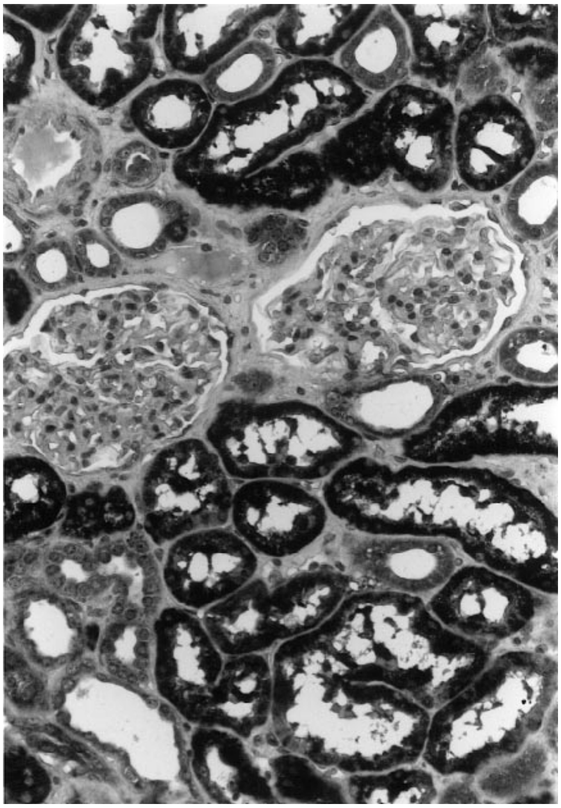

A

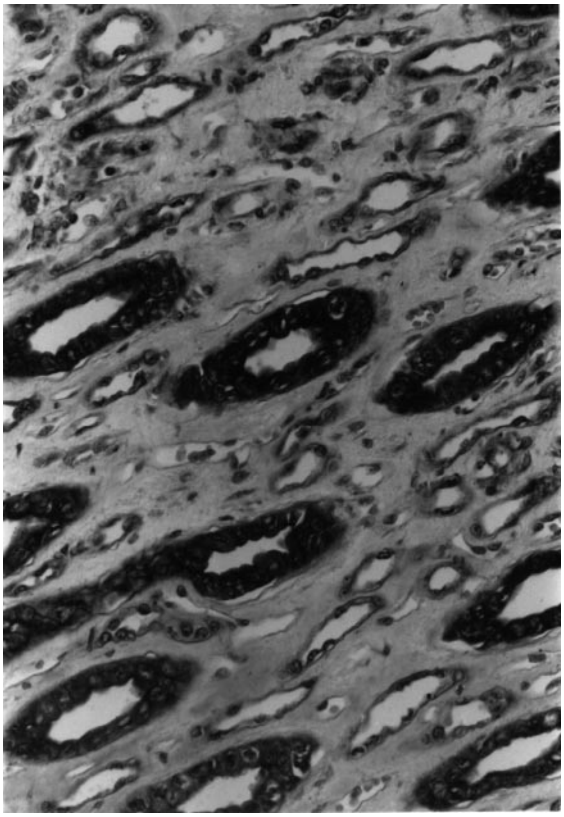

B

**Figure 2** Immunohistochemical localization of CYP3A in normal kidney: (**A**) renal cortex, (**B**) medulla. In the cortex there is strong CYP3A immunoreactivity in proximal tubular epithelial cells while there is no immunostaining in glomeruli. In the medulla there is strong immunoreactivity for CYP3A in collecting duct epithelium.

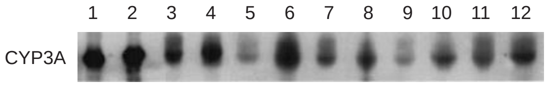

**Figure 3** Immunoblot of CYP3A of representative paired samples of normal kidney and kidney cancer. Lane 1 expressed CYP3A4 (10 µg of microsomal protein, 0.51 pmol of P450), lane 2 expressed CYP3A5 (2 µg of microsomal protein, 0.78 pmol of P450), lanes 3, 5, 7, 9 and 11 normal kidney; lanes 4, 6, 8, 10 and 12 corresponding kidney cancer samples (all clear cell carcinomas). A total of 30 µg of microsomal protein were loaded for each kidney sample

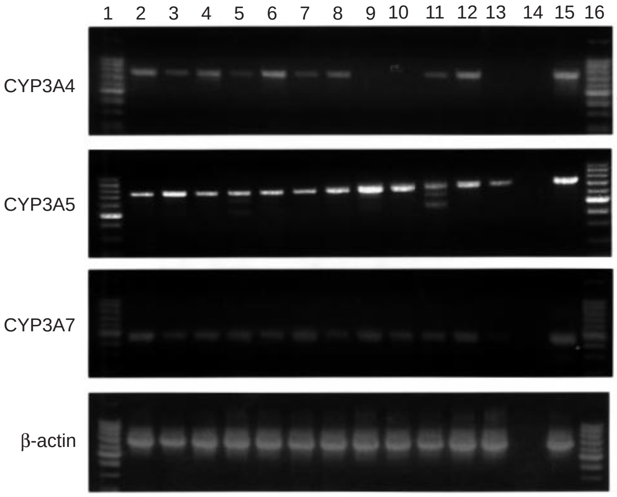

**Figure 4** Analysis of representative CYP3A4, CYP3A5, CYP3A7 and β-actin PCR products. Lanes 1 and 16 molecular size markers (100 bp ladder); lanes 2, 4, 6, 8, 10 and 12 normal kidney; lanes 3, 5, 7, 9, 11 and 13 corresponding kidney cancer samples (all clear cell carcinomas); lane 14 negative control and lane 15 positive control

### Immunoblotting

Immunoblotting of microsomes prepared from kidney tumour samples showed the presence of CYP3A in every tumour sample (Figure 3). Similarly, immunoblotting of microsomes from normal kidney also showed the presence of CYP3A in each sample of normal kidney. The intensity of the immunoreactive band in the tumours was at least as strong as that observed in the corresponding normal samples.

### RT-PCR

All the samples of both tumour and normal kidney amplified for β-actin and were subjected to PCR for CYP3A4, CYP3A5 and CYP3A7. CYP3A5 and CYP3A7 were consistently identified in all the tumour and normal samples (Figure 4). There was no apparent variation in the intensity of the amplified band observed in the corresponding normal and tumour samples for either CYP3A5 or CYP3A7. CYP3A4 mRNA was identified in 90% (18) of normal kidney samples and only in 65% (13) of the tumour samples. The tumours which did not show the presence of CYP3A4 mRNA were five clear cell carcinomas and the single papillary carcinoma. Both the normal samples in which CYP3A4 was not detected showed the presence of CYP3A4 in the corresponding tumour samples. Sequencing of the PCR products confirmed identity with the appropriate P450.

© Cancer Research Campaign 1999

### DISCUSSION

The human CYP3A P450 family consists of three closely related forms. CYP3A4 is the major form of P450 present in adult human liver, whereas CYP3A5 is only found in approximately 20–25% of livers (Schuetz et al, 1994). However, CYP3A5 appears to be more commonly present in extrahepatic tissues and has been identified in several normal tissues including colon (McKinnon et al, 1995), lung (Anttila et al, 1997), polymorphonuclear leucocytes (Janardan et al, 1996) and anterior pituitary gland (Murray et al, 1995) while CYP3A7 is the main form of P450 found in human fetal liver (Kitada and Kamataki, 1994). CYP3A can metabolize a variety of carcinogens (Gonzalez and Gelboin, 1994) and several anticancer drugs in clinical use, including ifosphamide (Chang et al, 1993; Walker et al, 1994) and paclitaxel (Harris et al, 1994). Thus there is potential involvement of CYP3A in both renal carcinogenesis and the response of established renal tumours to therapy.

CYP3A has previously been identified in various types of malignant tumours including breast cancer (Murray et al, 1993), colon cancer (Gervot et al, 1996), primary and secondary tumours of liver (Fritz et al, 1993), lung cancer (Kivistö et al, 1995*b*, 1996) and stomach cancer (Murray et al, 1998). The frequency of identification of CYP3A in the individual types of tumours has been different. However, the presence of CYP3A has not been previously investigated in renal tumours, although kidney tumours have been previously shown to express CYP1B1 (Murray et al, 1997) and microsomal epoxide hydrolase (McKay et al, 1995).

The individual members of the CYP3A family have almost identical molecular sizes and electrophoretic mobilities as judged by SDS-PAGE. Therefore, the strategy that was adopted to determine the presence and cellular localization of these forms of P450 in renal cell cancer and normal kidney was threefold: immunohistochemistry was used to identify the cellular localization of P450, immunoblotting to confirm the presence of immunoreactive CYP3A and RT-PCR to identify specifically CYP3A4, CYP3A5 and CYP3A7 mRNA. The CYP3A antibodies used in this study recognize CYP3A4, CYP3A5 and CYP3A7 and, because of their almost identical molecular size, it was not possible to resolve separate immunoreactive bands by immunoblotting. Thus RT-PCR was used to identify the presence of individual P450s of the CYP3A family. In each case the identity of the PCR product was confirmed by fluorescence-based sequencing.

Immunohistochemistry established that CYP3A was specifically localized to renal cancer cells. The direct demonstration by immunohistochemistry that CYP3A is expressed in tumour cells is important when considering the possible clinical consequences of CYP3A in renal tumours. The consistent expression of CYP3A in all renal tumours is at a much higher frequency than has been reported for the frequency of expression of CYP3A in some other types of tumour (Fritz et al, 1993; Kivistö et al, 1995*b*; Murray et al, 1998).

Previous studies have indicated that both CYP3A4 and CYP3A5 have been identified in normal kidney (Schuetz et al, 1992; Haehner et al, 1996) with CYP3A5 being present more frequently than CYP3A4 and possibly also quantitatively in larger amounts (Haehner et al, 1996). The consistent expression of CYP3A7 mRNA in the normal samples of kidney is at a much higher frequency than that observed by Haehner et al (1996) although CYP3A7 mRNA was frequently identified in normal adult lung tissue (Kivistö et al, 1996)

Renal cell cancer is considered to develop from proximal tubular epithelial cells (Motzer et al, 1996) and the presence of CYP3A in normal proximal tubular epithelium suggests that it may be involved in tumour development. The consistent expression of CYP3A in renal tumours is another indicator that this type of tumour expresses a phenotype associated with normal proximal tubular epithelium. Furthermore, the expression of individual forms of CYP3A in the kidney tumours suggest that these forms of P450 may contribute to the intrinsic multidrug resistance that is observed in this type of tumour (Chapman and Goldstein, 1995). The selection of anticancer drugs to treat kidney cancer should be considered in light of this study with avoidance of those agents which are significantly deactivated by these forms of P450. Paradoxically, because of the dual role of P450s in both activating and deactivating xenobiotics, the presence of CYP3A in kidney cancer may also provide molecular targets for certain anticancer drugs. One anticancer agent that is of particular interest is AQ4N, an alkylaminoanthroquinone, which is an inhibitor of both topoisomerase-I and topoisomerase-II (Patterson, 1993). This compound is activated by CYP3A and in hypoxic conditions, which are likely to exist in tumours, produces a cytotoxic metabolite of high potency, whereas in norm-oxic conditions, present in normal tissues, there is no cytotoxicity (Patterson, 1993). Use of this compound could be considered in the treatment of kidney cancer as all the tumours show significant expression of CYP3A.

### ACKNOWLEDGEMENTS

RTM was in receipt of a bursary from The Pathological Society of Great Britain and Ireland.

### REFERENCES

Anttila S, Hukkanen J, Hakkola J, Stjernvall T, Beaune P, Edwards RJ, Boobis AR, Pelkonen O and Raunio H (1997) Expression and localization of CYP3A4 and CYPA5 in human lung. *Am J Respir Cell Mol Biol* **16**: 242–249

Bradford M (1976) A rapid and sensitive method for the quantitation of microgram quantities of protein utilizing the principles of protein-dye binding. *Anal Biochem* **72**: 248–254

Capdevila J, Falck JR and Estabrook RW (1992) Cytochrome P450 and the arachidonate cascade. *FASEB J* **6**: 731–736

Chang TKH, Weber GF, Crespi CL and Waxman DJ (1993) Differential activation of cyclophosphamide and ifosphamide by cytochrome P450 2B and cytochrome P450 3A in human liver-microsomes. *Cancer Res* **53**: 5629–5637

Chapman AE and Goldstein LJ (1995) Multiple drug resistance; biologic basis and clinical significance in renal-cell carcinoma. *Semin Oncol* **22**: 17–28

Fritz P, Behrle E, Beaune P, Eichelbaum M and Kroemer HK (1993) Differential expression of drug metabolising enzymes in primary and secondary liver neoplasm: immunohistochemical characterization of cytochrome P450 3A and glutathione S-transferase. *Histochemistry* **99**: 443–451

Gervot L, Carrière V, Costet P, Cugnenc P-H, Berger A, Beaune PH and de Waziers I (1996) CYP3A5 is the major cytochrome P450 3A expressed in human colon and colonic cell lines. *Environ Toxicol Pharmacol* **2**: 381–388

Gonzalez FJ and Gelboin HV (1994) Role of human cytochromes P450 in the metabolic activation of chemical carcinogens and toxins. *Drug Metab Rev* **26**: 165–183

Guengerich FP (1992) Metabolic activation of carcinogens. *Pharmacol Ther* **54**: 17–62

Haehner BD, Gorski JC, Vandenbranden M, Wrighton SA, Janarden SK, Watkins PB and Hall SD (1996) Bimodal distribution of renal cytochrome P450 3A activity in humans. *Mol Pharmacol* **50**: 52–59

Harris JW, Rahman A, Kim BR, Guengerich FP and Collins JM (1994) Metabolism of taxol by human hepatic microsomes and liver slices: participation of cytochrome P450 3A4 and an unknown P450 enzyme. *Cancer Res* **54**: 4026–4035

© Cancer Research Campaign 1999

Janardan SK, Lown KS, Schiedlinrern P, Thummel KE and Watkins PB (1996) Selective expression of CYP3A5 and not CYP3A4 in human blood. *Pharmacogenetics* **6**: 379–385
Kawajiri K and Fujii-Kuriyama Y (1991) P450 and human cancer. *Jpn J Cancer Res* **82**: 1325–1335
Kitada M and Kamataki T (1994) Cytochrome P450 in human fetal liver: significance and fetal specific expression. *Drug Metab Dispos* **26**: 305–323
Kivistö KT, Kroemer HK and Eichelbaum M (1995*a*) The role of human cytochrome P450 enzymes in the metabolism of anticancer agents: implications for drug interactions. *Br J Clin Pharmacol* **40**: 523–530
Kivistö KT, Fritz P, Linder A, Friedel G, Beaune P and Kroemer HK (1995*b*) Immunohistochemical localization of cytochrome P450 3A in human pulmonary carcinomas and normal bronchial tissue. *Histochem Cell Biol* **103**: 25–29
Kivistö KT, Griese E-U, Fritz P, Linder A, Hakkola J, Raunio H, Beaune P and Kroemer HK (1996) Expression of cytochrome P450 3A enzymes in human lung: a combined RT-PCR and immunohistochemical analysis of normal tissue and lung tumours. *Arch Pharmacol* **353**: 207–212
Kovacs G, Akhtar M, Beckwith B, Gugert P, Cooper CS, Dleahunt B, Eble JN, Fleming S, Ljunberg B, Medeiros LJ, Moch H, Reuter VE, Ritz E, Roos G, Schmidt D, Srigley JR, Störkel S, Van Den Berg E and Zbar B (1997) The Heidelberg classification of renal cell tumours. *J Pathol* **183**: 131–133
McFadyen MCE, Melvin WT and Murray GI (1998) Regional distribution of individual forms of cytochrome P450 mRNA in normal human brain. *Biochem Pharmacol* **55**: 825–830
McKay JA, Weaver RJ, Murray GI, Ewen SWB, Melvin WT and Burke MD (1995) Expression of microsomal epoxide hydrolase in normal and neoplastic human kidney. *J Histochem Cytochem* **43**: 615–620
McKinnon RA, Burgess WM, Hall PdelaM, Roberts-Thomson SJ, Gonzalez FJ and McManus ME (1995) Characterisation of CYP3A gene subfamily expression in human gastrointestinal tissues. *Gut* **36**: 259–267
Massaad L, de Waziers I, Ribrag V, Janot F, Beaune PH, Morizet J, Gouyette A and Chabot GG (1992) Comparison of mouse and human colon tumors with regard to phase 1 and phase II drug-metabolizing enzyme systems. *Cancer Res* **52**: 6567–6575
Motzer RJ, Bander NH and Nanus DM (1996) Renal cell carcinoma. *N Engl J Med* **335**: 865–875
Murray GI, Barnes TS, Sewell HF, Ewen SWB, Melvin WT, Shaw PM, Fowler J and Burke MD (1987) Cytochrome P450 localisation in normal human adult and foetal liver by immunocytochemistry using a monoclonal antibody against human cytochrome P450. *Histochem J* **19**: 537–545
Murray GI, Barnes TS, Sewell HF, Ewen SWB, Melvin WT and Burke MD (1988) The immunocytochemical localisation and distribution of cytochrome P450 in normal human hepatic and extrahepatic tissues with a monoclonal antibody to human cytochrome P450. *Br J Clin Pharmacol* **25**: 465–475
Murray GI, Weaver RJ, Paterson PJ, Ewen SWB, Melvin WT and Burke M (1993) Expression of xenobiotic metabolising enzymes in breast cancer. *J Pathol* **169**: 347–353
Murray GI, Pritchard S, Melvin WT and Burke MD (1995) Cytochrome P450 CYP3A5 in the human anterior pituitary gland. *FEBS Letts* **364**: 79–82
Murray GI, Taylor MC, McFadyen MC, McKay JA, Greenlee WF, Burke MD and Melvin WT (1997) Tumour specific expression of cytochrome P450 CYP1B1. *Cancer Res* **57**: 3026–3031
Murray GI, Taylor MC, Burke MD and Melvin WT (1998) Enhanced expression of cytochrome P450 in stomach cancer. *Br J Cancer* **77**: 1040–1044
Nebert DW (1994) Drug-metabolizing-enzymes in ligand-modulated transcription. *Biochem Pharmacol* **47**: 25–37
Nelson DR, Koymans L, Kamataki T, Stegeman JJ, Feyereisen R, Waxman DJ, Waterman MR, Gotoh O, Coon JM, Estabrook RW, Gunsalus IC and Nebert DW (1996) P450 superfamily: update on new sequences, gene mapping, accession numbers and nomenclature. *Pharmacogenetics* **6**: 1–42
Patterson LH (1993) Rationale for the use of N-oxides of cytotoxic anthraquinones as prodrug DNA binding agents: a new class of bioreductive drugs. *Cancer Metast Rev* **12**: 119–234
Roberts-Thomson SJ, McManus ME, Tukey RH, Gonzalez FJ and Holder GM (1995) Metabolism of polycyclic aza-aromatic carcinogens catalyzed by four expressed human cytochromes P450. *Cancer Res* **55**: 1052–1059
Sarabia SF, Zhu BT, Kurosawa T, Tohma M and Liehr JG (1997) Mechanism of cytochrome P450-catalyzed aromatic hydroxylation of estrogens. *Chem Res Toxicol* **10**: 767–771
Schuetz EG, Schuetz JD, Grogan WM, Naray-Fejes-Toth A, Fejes-Toth G, Raucy J, Guzelian P, Gionela K and Watlington CO (1992) Expression of cytochrome P450 3A in amphibian, rat, and human kidney. *Arch Biochem Biophys* **294**: 206–214
Schuetz JD, Beach DL and Guzelian PS (1994) Selective expression of cytochrome P450 CYP3A mRNAs in embryonic and adult human liver. *Pharmacogenetics* **4**: 11–20
Shaw PM, Barnes TS, Cameron D, Engeset J, Melvin WT, Omar G, Petrie JC, Rush WR, Snyder CP, Whiting PH and Burke MD (1989) Purification and characterization of an anticonvulsant-induced human cytochrome P450 catalysing cyclosporin metabolism. *Biochem J* **262**: 653–663
Stadler W and Vogelzang NJ (1993) Human renal cancer carcinogenesis; a review of recent advances. *Annal Oncol* **4**: 451–462
Walker D, Flinois JP, Monkman SC, Beloc C, Boddy AV, Cholerton S, Daly AK, Lind MJ, Pearson ADJ, Beaune PH and Idle JR (1994) Identification of the major human hepatic cytochrome P450 involved in activation and n-dechloroethylation of ifosfamide. *Biochem Pharmacol* **47**: 1157–1160
Yagoda A, Abi-Rached B and Petrylak D (1995) Chemotherapy for advanced renal cell carcinoma. *Semin Oncol* **22**: 42–60

 *© Cancer Research Campaign 1999*
